# Direct measurement of a patient's entrance skin dose during pediatric cardiac catheterization

**DOI:** 10.1093/jrr/rru050

**Published:** 2014-06-26

**Authors:** Lue Sun, Yusuke Mizuno, Mari Iwamoto, Takahisa Goto, Yasuhiro Koguchi, Yuka Miyamoto, Koji Tsuboi, Koichi Chida, Takashi Moritake

**Affiliations:** 1 Graduate School of Comprehensive Human Sciences, University of Tsukuba, 1–1–1 Tennodai, Tsukuba, Ibaraki 305–8575, Japan; 2 Department of Anesthesiology, Yokohama City University, 3–9 Fukuura, Kanazawa-ku, Yokohama, Kanagawa 236–0004, Japan; 3 Department of Pediatrics, Yokohama City University, 3–9 Fukuura, Kanazawa-ku, Yokohama, Kanagawa 236–0004, Japan; 4 Oarai Research Center, Chiyoda Technol Corporation, 3681 Naritacho, Oarai-machi, Higashiibaraki-gun, Ibaraki 311–1313, Japan; 5 Proton Medical Research Center, Faculty of Medicine, University of Tsukuba, 1–1–1 Tennodai, Tsukuba, Ibaraki 305–8575, Japan; 6 Department of Radiological Technology, School of Health Sciences, Faculty of Medicine, Tohoku University, 2–1 Seiryo-machi, Aoba-ku, Sendai, Miyagi 980–8575, Japan; 7 Department of Radiological Health Science, Institute of Industrial Ecological Sciences, University of Occupational and Environmental Health, Japan, 1–1 Iseigaoka, Yahatanishi-ku, Kitakyushu, Fukuoka 807–8555, Japan

**Keywords:** cardiac catheterization, entrance skin dose, pediatric heart disease, dosimetry

## Abstract

Children with complex congenital heart diseases often require repeated cardiac catheterization; however, children are more radiosensitive than adults. Therefore, radiation-induced carcinogenesis is an important consideration for children who undergo those procedures. We measured entrance skin doses (ESDs) using radio-photoluminescence dosimeter (RPLD) chips during cardiac catheterization for 15 pediatric patients (median age, 1.92 years; males, *n* = 9; females, *n* = 6) with cardiac diseases. Four RPLD chips were placed on the patient's posterior and right side of the chest. Correlations between maximum ESD and dose–area products (DAP), total number of frames, total fluoroscopic time, number of cine runs, cumulative dose at the interventional reference point (IRP), body weight, chest thickness, and height were analyzed. The maximum ESD was 80 ± 59 (mean ± standard deviation) mGy. Maximum ESD closely correlated with both DAP (*r* = 0.78) and cumulative dose at the IRP (*r* = 0.82). Maximum ESD for coiling and ballooning tended to be higher than that for ablation, balloon atrial septostomy, and diagnostic procedures. In conclusion, we directly measured ESD using RPLD chips and found that maximum ESD could be estimated in real-time using angiographic parameters, such as DAP and cumulative dose at the IRP. Children requiring repeated catheterizations would be exposed to high radiation levels throughout their lives, although treatment influences radiation dose. Therefore, the radiation dose associated with individual cardiac catheterizations should be analyzed, and the effects of radiation throughout the lives of such patients should be followed.

## INTRODUCTION

The incidence of congenital cardiac diseases is ∼1% [1]. Cardiac catheterization is an essential procedure for precise diagnosis and/or treatment for congenital cardiac diseases. However, complex cardiac catheterization procedures result in higher radiation doses due to a longer fluoroscopy time, and congenital cardiac diseases often require repeated catheterization procedures for staged operations and for their follow-ups, resulting in a large cumulative lifetime radiation dose per individual. Meanwhile, due to advancements in treatment technique in recent years, the number of long-term survivors is increasing. Therefore, reducing radiation doses during pediatric cardiac catheterizations is an important issue with respect to improving the quality of life for survivors.

The risks of radiation-induced late effect, such as leukemia and solid cancer, are greater in children than in adults [2, 3]. Among different sites and types of cancer, the risk of developing non-melanoma skin cancer and thyroid cancer is much higher in children relative to adults among those exposed to atomic bomb radiation [3]. Therefore, unnecessary exposure of the thyroid or skin to radiation should be avoided as much as possible in young patients undergoing cardiac catheterizations. Furthermore, it is necessary to reduce the area exposed, as the heart to body ratio is much greater in a child than in an adult. However, only a few studies have investigated radiation exposure and its distribution during pediatric catheterizations for congenital heart disease [4–6]. Thus, to increase the safety of interventional procedures for children, the lifetime total entrance skin dose (ESD) and its geometric distribution should be measured in individuals.

**Fig. 1.**
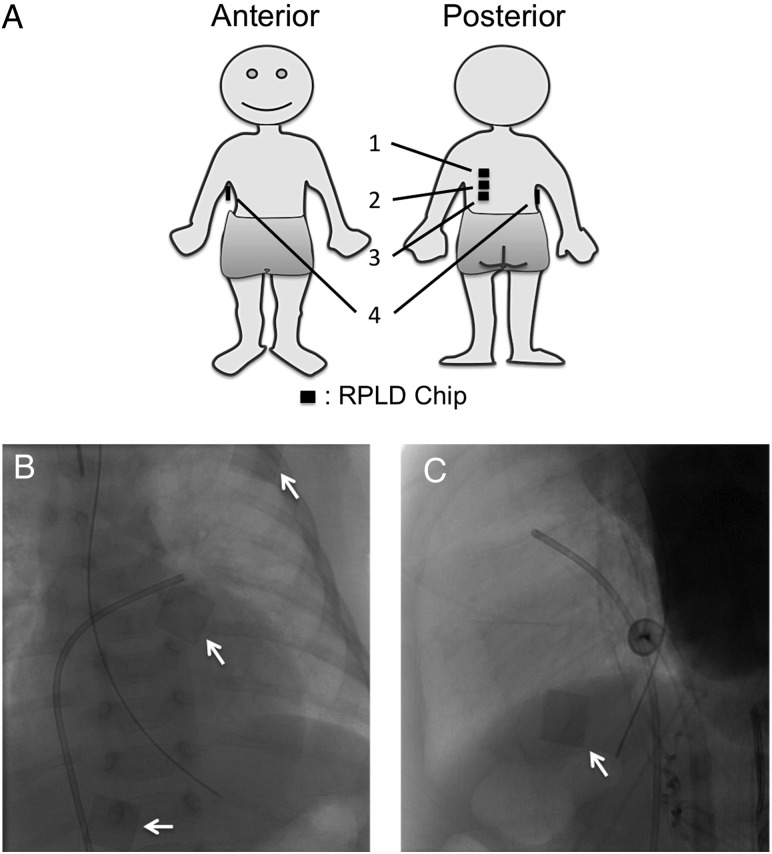
Radio-photoluminescence dosimeter (RPLD) chips were placed on the posterior chest and right lateral chest. **(A**) Schema of the site of the RPLD chips. The number shows the position number for RPLD placement. (**B**) Typical angiogram (PA view). (**C**) Typical angiogram (RL view). Arrows indicate RPLD chips.

A radio-photoluminescence dosimeter (RPLD) has been used for direct measurement of patients' ESD, because RPLD is sensitive to X-rays with sufficient accuracy through a wide range (10 μGy to 500 Gy) of detection and because it can be placed at many points on the skin surface due to its small size and radiolucency under fluoroscopy and cine run (Fig. 1B and C) [7–12]. Therefore, this study evaluated the ESD and its distribution using RPLD chips for patients with congenital heart diseases who were treated via catheterization. In addition, we analyzed the correlation between the maximum ESD and several angiographic parameters and body parameters so as to easily predict maximum ESD in future cases without the need for ESD measurements using RPLD.

## MATERIALS AND METHODS

### Patients

ESD dosimetry was performed in 15 cases from March to December 2008 (Table 1). The patients were selected at random. The patients were comprised of nine males and six females with a median age of 1 year and 11 months old (range, 1 month to 11 years and 11 months) and a median weight of 9 kg (range, 3 kg to 48 kg).

**Table 1.** Characteristics of the pediatric patients in our study

Case	Age	Weight (kg)	Height (cm)	Chest thickness (mm)	Sex	Disease	Procedure	Number of cardiac catheterizations (follow-up period)
1	8 y 6 m	31	128	160	M	PDA	PDA coiling	1 (5 y 7 m)
2	0 y 1 m	3	50	85	M	PA/IVS Ebstein	BAS	5 (5 y 1 m)
3	0 y 11 m	8	71	90	M	TGA	PA ballooning	5 (5 y 9 m)
4	2 y 2 m	9	79	102	F	DORV	Diagnosis	6 (7 y 1 m)
5	1y 11 m	11	86	106	M	TOF	PA ballooning	5 (6 y 11 m)
6	0 y 3 m	4	53	no data	M	AoC, VSD, PS	Diagnosis	3 (3 y 7 m)
7	1 y 9 m	9	78	106	M	DORV	PA ballooning	6 (6 y 8 m)
8	2 y 6 m	10	81	140	F	DORV	PA ballooning	12 (9 y 5 m)
9	2 y 3 m	10	85	112	F	PA/IVS	PA ballooning	10 (7 y 1 m)
10	2 y 10 m	13	95	115	M	cTGA	Aortopulmonary collateral vessels coiling	8 (7 y 6 m)
11	0 y 1 m	3	50	83	M	HLHS	BAS	9 (4 y 8 m)
12	0 y 7 m	8	69	87	F	PS, AS	PA ballooning	5 (5 y 0 m)
13	0 y 10 m	10	75	98	F	DORV	Coiling	3 (5 y 5 m)
14	11 y 11 m	48	150	129	F	WPW	Ablation	1 (5 y 10 m)
15	0 y 3 m	4	50	no data	M	HLHS	Diagnosis	9 (4 y 8 m)

Case 11 and Case 15 are the same patient. AoC = aortic coarctation, AS = aortic stenosis, BAS = balloon atrial septostomy, cTGA = corrected transposition of great arteries, DORV = double-outlet right ventricle, HLHS = hypoplastic left heart syndrome, PA ballooning = pulmonary artery ballooning, PA/IVS = pulmonary atresia with intact ventricular septum, PDA = patent ductus arteriosus, PDA coiling = patent ductus arteriosus coiling, PS = pulmonary stenosis, TOF = Tetralogy of Fallot, VSD = ventricular septal defect, WPW = Wolff–Parkinson–White syndrome.

This study was conducted in accordance with the Declaration of Helsinki. All protocols were approved by Yokohama City University Hospital (Yokohama, Japan). Written informed consent was obtained from patients' parents before commencing procedures.

### Angiogram technique

A biplane X-ray imaging system equipped with two image intensifiers and a digital cine mode (INFX-8000 V/JB, Toshiba, Japan) was used. The X-ray fluoroscopic unit was used with the following conditions: source-to-image-intensifier distances of 90 cm for frontal view and 110 cm for lateral view, an image intensifier field size of 12.7 cm, a tube potential of 75 kVp, and an acquisition rate of 7.5–30 frames per second for pulsed fluoroscopy. A 0.3-mm thick copper filter was added for fluoroscopy. For cine angiography, a 1.8-mm thick aluminum filter was inserted automatically.

Dose–area product (DAP), total number of frames, total fluoroscopic time, number of cine runs, cumulative dose at the interventional reference point (IRP), body weight, chest thickness, and height were recorded. Among these values, DAP, total number of frames, and total fluoroscopic time were total values for both planes (total value = frontal plane value + lateral plane value). We also used the double product of body parameter and total fluoroscopic time, which are the weight–total fluoroscopic time product, the chest thickness–total fluoroscopic time product, and the height–total fluoroscopic time product.

### Dosimetry technique

Patient ESD was measured using small RPLD chips (Chiyoda Technol Co., Tokyo, Japan). The RPLD chip is 8-mm square and 1-mm thick with sufficient radiolucency for fluoroscopy. Each chip was wrapped in a thin plastic film in order to avoid staining from sweat or blood during the interventional procedures. Three wrapped RPLD chips were placed on the posterior chest (Positions Nos 1, 2 and 3 in Fig. 1A), and one wrapped RPLD chip was placed on the right lateral chest (Position No. 4 in Fig. 1A).

The dose response of RPLD is susceptible to the X-ray energy at the range normally used for angiography (30–40 keV). Therefore, an average calibration factor to adjust the RPLD readout value to the ESD was calculated and then used as described previously [9, 12].

### Statistical analysis

Correlations between maximum ESD and DAP, total number of frames, total fluoroscopic time, number of cine runs, cumulative dose at the IRP, weight, chest thickness, and height were analyzed using linear regression. Student's *t*-test was used to compare the two procedure groups. Statistical significance was defined as a *P*-value of less than 0.05.

## RESULTS

The patients' age, weight, height, chest thickness, sex, disease, interventional radiology procedure, and number of cardiac catheterizations are summarized in Table 1. Three patients underwent cardiac catheterization for diagnostic purposes. The remaining 12 patients underwent interventional radiology (therapeutic) procedures. The therapeutic procedures were divided into four groups: coil embolization, balloon dilatation, ablation, and balloon atrial septostomy (BAS). Of the 15 patients, 13 underwent multiple cardiac catheterizations during the observation period.

Table 2 summarizes maximum ESD (the largest ESD value recorded from the four RPLD chips put on the patient's skin), DAP, total number of frames, total fluoroscopic time, number of cine runs, and cumulative dose at the IRP of patients.

**Table 2.** Maximum ESDs and angiographic parameters in our study

Case	Maximum ESD (mGy)	DAP (cGy × cm^2^)	Total number of frames	Total fluoroscopic time (s)	Number of cine runs	Cumulative dose at the IRP (Gy)
1	71	3 208.9	1 676	1 467	19	0.2
2	66	1 018.6	1 076	7 150	13	0.1
3	150	2 875.7	5 873	3 875	35	0.3
4	50	586.1	1 963	1 077	16	0.1
5	140	3 119.5	4 342	4 132	44	0.3
6	34	598.0	1 242	3 167	16	0.1
7	49	1 571.8	1 948	1 534	24	0.1
8	100	3 124.8	2 702	4 356	35	0.2
9	44	2 704.9	1 873	1 952	16	0.1
10	210	8 627.3	2 998	5 390	35	0.4
11	12	145.7	231	1 334	8	0.0
12	160	2 766.2	4 314	4 166	50	0.3
13	66	2 832.7	1 627	3 967	25	0.2
14	23	2 364.1	50	3 530	14	0.3
15	26	1 436.9	801	806	12	0.1
Average	80	2 465.4	2 181.1	3 193.5	24.1	0.2
SD	59	2 003.5	1 623.5	1 807.6	12.8	0.1

**Fig. 2.**
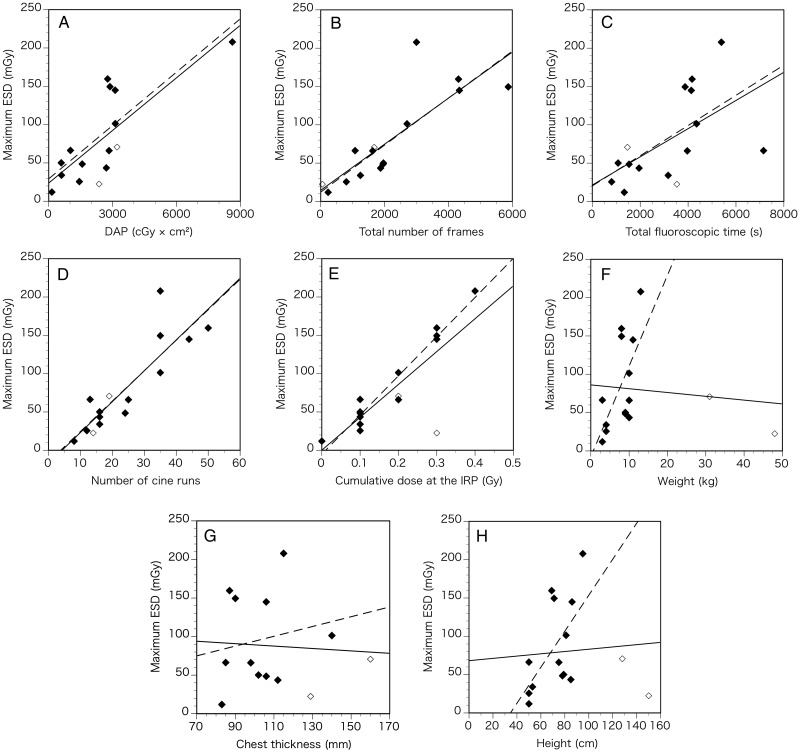
Correlations between maximum entrance skin dose (ESD) and angiographic and body parameters. Closed diamonds indicate patients younger than 3 years; open diamonds indicate patients older than 3 years. Solid lines on the graphs indicate linear regressions for all patients; dashed lines on the graphs indicate linear regressions for patients younger than 3 years. (**A**) Correlation between maximum ESD and dose–area product (DAP). (**B**) Correlation between maximum ESD and total number of frames. (**C**) Correlation between maximum ESD and total fluoroscopic time. (**D**) Correlation between maximum ESD and number of cine runs. (**E**) Correlation between maximum ESD and cumulative dose at the interventional reference point (IRP). (**F**) Correlation between maximum ESD and weight. (**G**) Correlation between maximum ESD and chest thickness. (**H**) Correlation between maximum ESD and height.

We investigated the relationship between maximum ESD and angiographic parameters (Fig. 2). DAP (*r* = 0.78, *P* < 0.001), total number of frames (*r* = 0.83, *P* < 0.001), number of cine runs (*r* = 0.87, *P* < 0.001), and cumulative dose at the IRP (*r* = 0.82, *P* < 0.001) showed good correlations with maximum ESD, whereas total fluoroscopic time poorly correlated with maximum ESD (*r* = 0.56, *P* = 0.029) (Fig. 2A–E, Table 3). Body weight (*r* = −0.10, *P* = 0.721), chest thickness (*r* = −0.06, *P* = 0.846), and height (*r* = 0.07, *P* = 0.801) did not correlate with maximum ESD (Fig. 2F–H, Table 3).

**Table 3.** Linear regression lines in Fig. 2

Outcome variable (y)	Predictor variable (x)	Regression line	Correlation coefficient (*r*)	Coefficient of determination (*r*^2^)	*P*-value	Remarks
Maximum ESD (mGy)	DAP (cGy × cm^2^)	*y* = 0.0229*x* + 23.7509	0.78	0.60	< 0.001	Solid line on Fig. 2A
		*y* = 0.0232*x* + 29.2553	0.81	0.66	< 0.001	Dashed line on Fig. 2A
	Total number of frames	*y* = 0.0300*x* + 14.6284	0.83	0.68	< 0.001	Solid line on Fig. 2B
		*y* = 0.0306*x* + 12.2695	0.81	0.66	< 0.001	Dashed line on Fig. 2B
	Total fluoroscopic time (s)	*y* = 0.0184*x* + 21.4068	0.56	0.32	0.029	Solid line on Fig. 2C
		*y* = 0.0197*x* + 20.2870	0.60	0.37	0.029	Dashed line on Fig. 2C
	Number of cine runs	*y* = 4.0267*x* − 17. 0306	0.87	0.76	< 0.001	Solid line on Fig. 2D
		*y* = 3.9705*x* − 15.1770	0.86	0.74	< 0.001	Dashed line on Fig. 2D
	Cumulative dose at the IRP (Gy)	*y* = 428.9060*x* + 0.0842	0.82	0.67	< 0.001	Solid line on Fig. 2E
		*y* = 511.2453*x* − 5.1434	0.97	0.94	< 0.001	Dashed line on Fig. 2E
	Weight (kg)	*y* = −0.4965*x* + 86.1373	−0.10	0.01	0.721	Solid line on Fig. 2F
		*y* = 11.8366*x* − 7.5641	0.63	0.40	0.020	Dashed line on Fig. 2F
	Chest thickness (mm)	*y* = −0.1565*x* + 104.8996	−0.06	0.00	0.846	Solid line on Fig. 2G
		*y* = 0.6344*x* + 30.5759	0.17	0.03	0.613	Dashed line on Fig. 2G
	Height (cm)	*y* = 0.1492*x* + 68.2127	0.07	0.01	0.801	Solid line on Fig. 2H
		*y* = 2.3502*x* − 81.3748	0.59	0.35	0.032	Dashed line on Fig. 2H

After eliminating two outlier cases with ages older than 3 years (Cases 1 and 14 in Tables 1 and 2), the correlation coefficient (*r*) between maximum ESD and body weight and height markedly increased to 0.63 (*P* = 0.020) (Fig. 2F, Table 3) and 0.59 (*P* = 0.032) (Fig. 2H, Table 3), respectively. In contrast, the correlation coefficients between maximum ESD and DAP (*r* = 0.81, *P* < 0.001), total number of frames (*r* = 0.81, *P* < 0.001), total fluoroscopic time (*r* = 0.60, *P* = 0.029), number of cine runs (*r* = 0.86, *P* < 0.001), cumulative dose at the IRP (*r* = 0.97, *P* < 0.001) and chest thickness (*r* = 0.17, *P* = 0.613) did not change so much (Fig. 2A–E and G, Table 3).

**Fig. 3.**
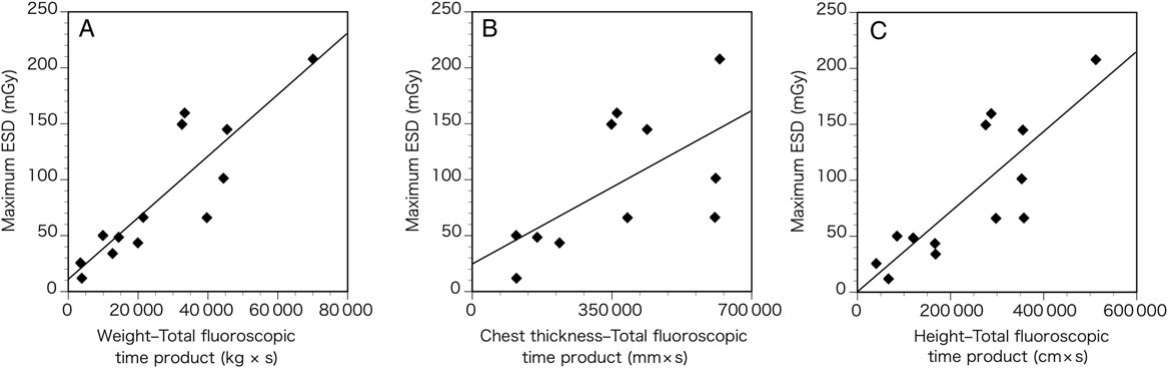
Correlations between maximum ESD and double products of body parameter and total fluoroscopic time for patients younger than 3 years. (**A**) Correlation between maximum ESD and weight–total fluoroscopic time product. (**B**) Correlation between the maximum ESD and chest thickness–total fluoroscopic time product. (**C**) Correlation between the maximum ESD and height–total fluoroscopic time product.

When the DAP meter is not equipped with a fluoroscopy unit, or when the cumulative dose at the IRP cannot be displayed, the only total fluoroscopic time is available in real-time during catheterization. Therefore, we investigated the relationship between maximum ESD and the double product of body parameters (i.e. body weight, chest thickness, and height) and total fluoroscopic time after elimination of the two outlier cases. The correlation coefficients for weight–total fluoroscopic time product, the chest thickness–total fluoroscopic time product, and the height–total fluoroscopic time product were 0.88 (*P* < 0.001), 0.62 (*P* = 0.041), and 0.83 (*P* < 0.001), respectively (Fig. 3, Table 4), which are much higher than the values without the double product.

**Table 4.** Linear regression lines in Fig. 3

Outcome variable (*y*)	Predictor variable (*x*)	Regression line	Correlation coefficient (*r*)	Coefficient of determination (*r*^2^)	*P*-value	Remarks
Maximum ESD (mGy)	Weight–total fluoroscopic time product (kg × s)	*y* = 0.0028*x* + 10.9535	0.88	0.77	< 0.001	Solid line on Fig. 3A
	Chest thickness–total fluoroscopic time product (mm × s)	*y* = 0.0002*x* + 24.6247	0.62	0.39	0.041	Solid line on Fig. 3B
	Height–total fluoroscopic time product (cm × s)	*y* = 0.0004*x* + 0.3735	0.83	0.68	< 0.001	Solid line on Fig. 3C

**Fig. 4.**
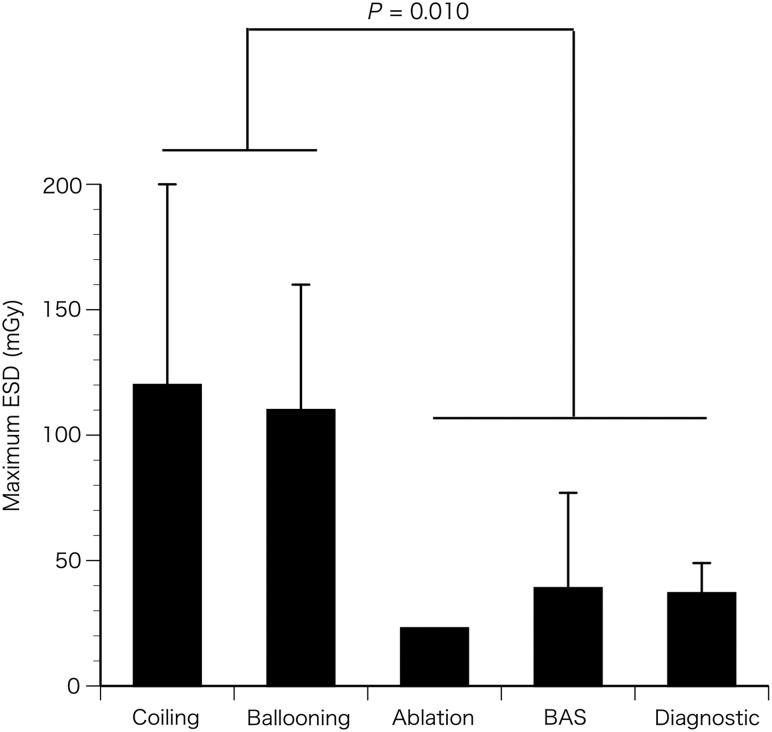
Maximum ESD in different procedures. Data indicate mean + standard deviation (SD).

Figure 4 shows the maximum ESD for different procedures. Maximum ESD (mean ± standard deviation) in the group undergoing coiling (120 ± 80 mGy, *n* = 3) or ballooning (110 ± 50 mGy, *n* = 6) tended to be higher than that of patients undergoing ablation (23 mGy, *n* = 1), BAS (39 ± 38 mGy, *n* = 2) or diagnostic angiography (37 ± 12 mGy, *n* = 3) (*P* = 0.010).

**Fig. 5.**
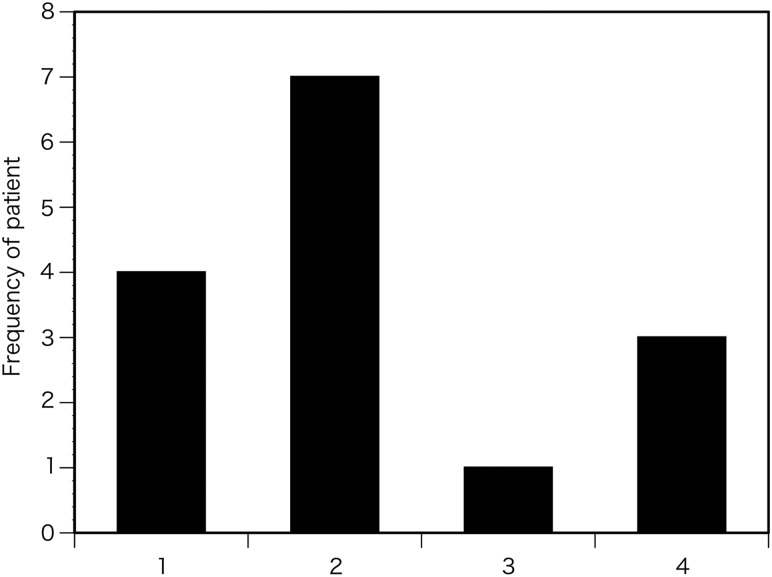
Number of patients who showed the maximum ESD at each position. The number on the horizontal axis shows the position number for RPLD placement as indicated in Fig. 1A.

As for the dose distribution, the number of patients who showed the maximum ESD in RPLD Position Nos1, 2, 3 and 4 were four, seven, one and three, respectively (Fig. 5).

## DISCUSSION

Reducing unnecessary X-ray exposure during cardiac catheterization is of great importance for pediatric patients, because these patients have high tissue susceptibility to ionizing radiation [13]. For medical exposure, the maximum ESD should not exceed the threshold dose that causes the deterministic effect. However, the largest value for the maximum ESD in this study was 210 mGy (Case 10 in Table 2), which is far below the 2-Gy threshold dose for temporary erythema [14]. In fact, skin injury did not occur after the procedure.

As for the stochastic effect, a previous study of Hiroshima and Nagasaki atomic bomb survivors indicated that children younger than 20 years are more susceptible than adults to induction of cancers [15]. Moreover, Mathews *et al*. showed that undergoing CT scans during childhood and adolescence is followed by an increase in cancer incidence for all cancers combined and for many individual types of cancer [16]. Sasaki *et al*. showed that mice exposed to radiation while in infancy had a significantly decreased lifespan and an increase in the incidence of cancer when compared with mice exposed as adults [17]. These observations suggest that radiation-induced cancer risks in children who undergo cardiac catheterization are likely to be higher than those for adults. In particular, the risk of radiation-induced skin and thyroid cancer is higher in younger (10 years of age) than in older patients (50 years of age) based on an analysis of site-specific sex-averaged excess relative risk and excess absolute rate attained at age 70 after atomic bomb radiation exposure [3]. According to their report, interventional procedures for congenital heart disease could be associated with higher risks for solid cancer, because the thyroid is located much closer to the heart in children than in adults and because skin irradiation is inevitable when patients require X-ray fluoroscopy. Furthermore, when a small child is treated with the same size of the field exposed, the totally irradiated body fraction is larger when compared with that of an adult, which means an increase in the effective dose for children during cardiac catheterization.

DAP is defined as the absorbed dose multiplied by the area irradiated; therefore, DAP reflects not only the dose within the radiation field but also the area of tissue irradiated. Bacher *et al*. reported a good correlation (*r* = 0.90) between peak ESD and DAP in pediatric catheterization [14]. We also found a good correlation (*r* = 0.78) between maximum ESD and DAP in this study (Fig. 2A, Table 3). Among the parameters analyzed, DAP could be a useful indicator for predicting the maximum ESD, since it has the advantages of being easily measured in real time via the permanent installation of a DAP meter on the X-ray fluoroscopic unit. For the same reason, it would be useful in clinical settings to have an indicator of the cumulative dose at the IRP (*r* = 0.82) (Fig. 2E, Table 3), which is located on the central ray of the X-ray beam, 15 cm from the isocenter toward the focal spot.

Chida *et al*. reported a good correlation between DAP and weight in cardiac catheterization for both the diagnostic (*r* = 0.819) and the therapeutic (*r* = 0.895) procedures in children; this is because the X-ray output to the larger patients is usually greater [2]. According to this theory, the maximum ESD is a strong determinant of DAP and should correlate with body parameters, such as weight, chest thickness, and height. However, we found no correlation between maximum ESD and those parameters (weight, *r* = −0.10; chest thickness, *r* = −0.06; height, *r* = 0.07) (Fig. 2F–H, Table 3). One explanation for our results is that outlier cases with large body size were included in our study. Indeed, Chida *et al*. reported a poor correlation between weight and radiation dose for adult patients [2, 18, 19]. When the two outliers (Cases 1 and 4 on Table 1) were eliminated, the correlation coefficient between maximum ESD and weight and height increased to 0.63 (Fig. 2F, Table 3) and 0.59 (Fig. 2H, Table 3), respectively. Hence body weight and height could correlate with maximum ESD for children younger than 3 years.

The correlation coefficients between maximum ESD and double products of body parameter–total fluoroscopic time products were much higher than those for body parameters alone (Fig. 3, Table 4). Total fluoroscopic time and body parameters are always available in real time; therefore, when DAP or cumulative dose at the IRP cannot be monitored during the procedure, body parameter–fluoroscopic time products could be a surrogate indicator for the maximum ESD during cardiac catheterization for pediatric patients younger than 3 years.

Maximum ESDs for coiling and ballooning procedures tended to be higher (*P* = 0.010) than that for ablation, BAS and diagnostic procedures (Fig. 4). This trend is probably related to the fact that coiling and ballooning techniques require longer procedural times, as previously reported [2]. In our study, this trend in maximum ESD was more similar to the trend in total number of frames (Suppl. Fig. 1B) and number of cine runs (Suppl. Fig. 1D) than to total fluoroscopic time (Suppl. Fig. 1C), which is probably because the maximum ESD was more affected in cine than in fluoroscopy. Thus, avoidance of unnecessary cine runs, especially during coiling and ballooning procedures, is warranted.

The maximum ESD was distributed at around the upper posterior chest (Position Nos 1 and 2 in Fig. 1A) (Fig. 5). Three patients (20%) showed maximum ESD in the right lateral chest (Position No. 4 in Fig. 1A) (Fig. 5). A previous report demonstrated a wide variation in the location of maximum ESD after cardiac interventional radiology procedures in adults [10]. Our results also showed that the maximum ESD spread in the posterior and lateral chest during pediatric cardiac catheterization (Fig. 5); therefore, the radiation dose as well as its location must be evaluated for each procedure.

There are some limitations in this study. One limitation is the small number (*n* = 4) of RPLD chips placed on the patient to represent the whole area exposed to X-rays. Another limitation is that the statistical analysis of the small number of patients (*n* = 15) contains high uncertainty. Therefore, we are now developing a jacket equipped with an RPLD chip that can obtain more precise dose distributions. This system will provide us with a better understanding of the radiation dose necessary to reduce the risk of late effects from pediatric interventional radiology.

In this study, we measured the maximum ESD using RPLD chips and analyzed its correlation with factors related to cardiac catheterization in children. DAP and cumulative dose at the IRP were found to be useful parameters for predicting the maximum ESD in real time during the procedure. When neither DAP nor the IRP can be monitored, the body parameter–total fluoroscopic time product, especially weight–total fluoroscopic time product and height–total fluoroscopic time, could be a useful surrogate indicator of the maximum ESD for children younger than 3 years. The maximum ESD was strongly influenced by the treatment procedure; therefore, children who need repeated catheterization for complex congenital heart disease should undergo measurement of their total radiation doses and its location through their lifespans.

## SUPPLEMENTARY DATA

Supplementary data is available at the *Journal of Radiation Research* online.

## FUNDING

This research is partly supported by institutional sources and in part by Grants-in-Aid (Nos 21611012 and 24300179) from the Ministry of Education, Science, Sports and Culture of Japan. Funding to pay the Open Access publication charges for this article was provided by Grants-in-Aid (No. 26293135) from the Ministry of Education, Science, Sports and Culture of Japan.

## Supplementary Material

Supplementary Data
