# Supplementary material for: Direct measurement of a patient's entrance skin dose during pediatric cardiac catheterization
Source: J Radiat Res. 2014 Jun 26;55(6):1122–30. doi: 10.1093/jrr/rru050 (PMC4229915; doi:10.1093/jrr/rru050)
Supplement: Supplementary Data [file supp_55_6_1122__index.html]

Direct measurement of a patient's entrance skin dose during pediatric cardiac catheterization — Supplementary Data 

# Direct measurement of a patient's entrance skin dose during pediatric cardiac catheterization

## Supplementary Data

Supplementary Data

**Files in this Data Supplement:**

- Supplementary Figure 1 - pdf file
- Supplementary Data - Doc file
