# Supplementary figures and images for: Direct measurement of a patient's entrance skin dose during pediatric cardiac catheterization
Source: J Radiat Res. 2014 Jun 26;55(6):1122–30. doi: 10.1093/jrr/rru050 (PMC4229915; doi:10.1093/jrr/rru050)

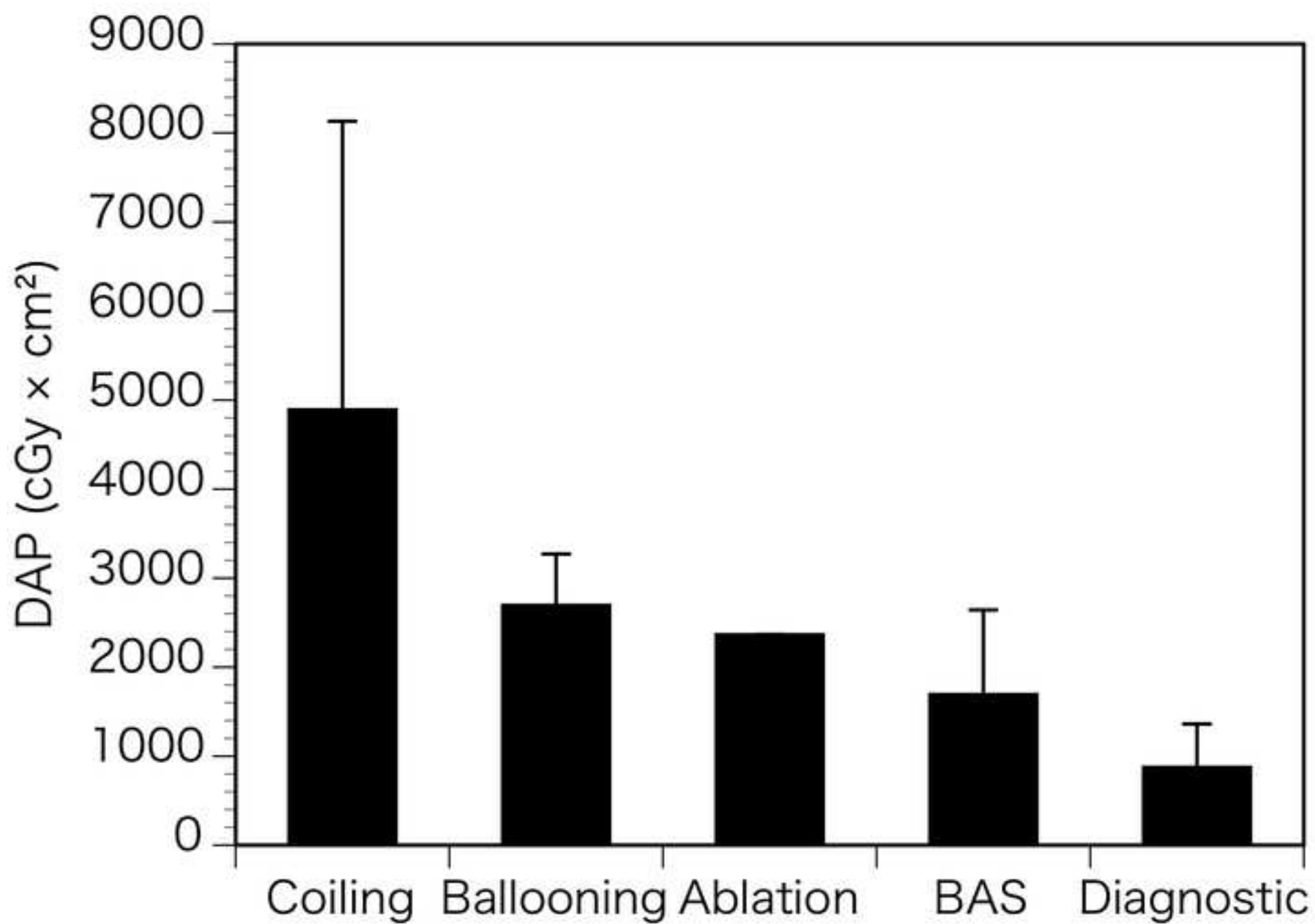

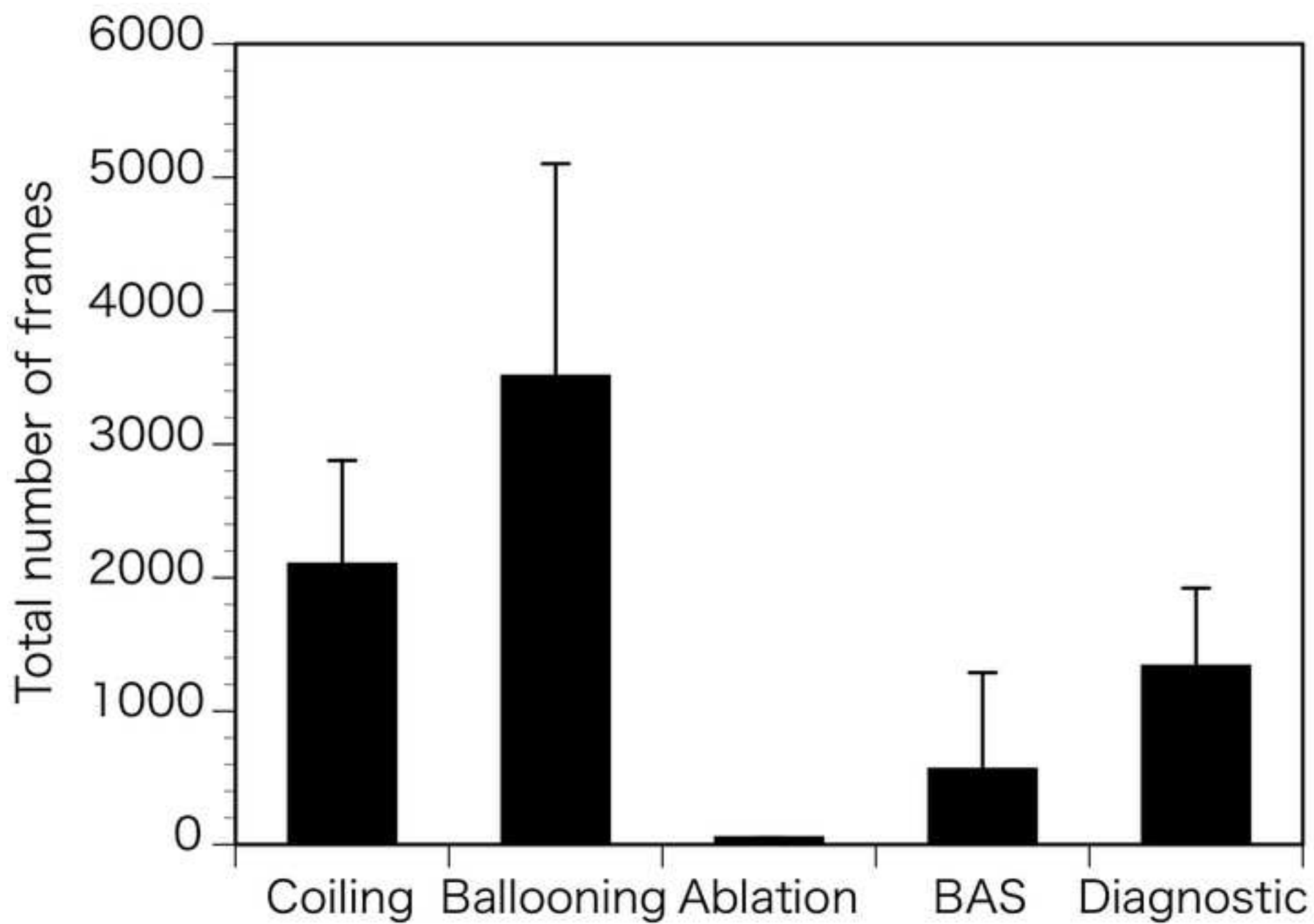

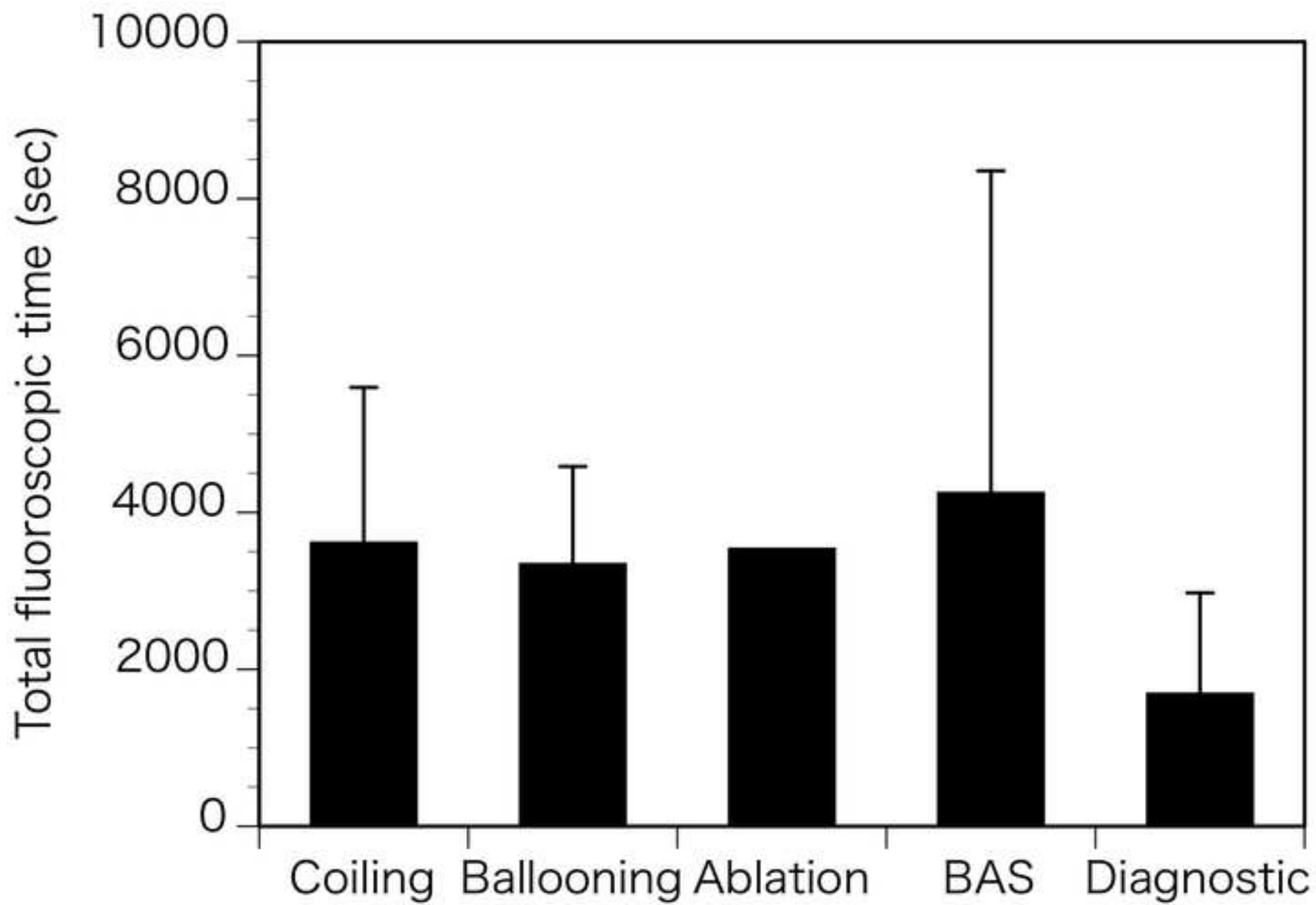

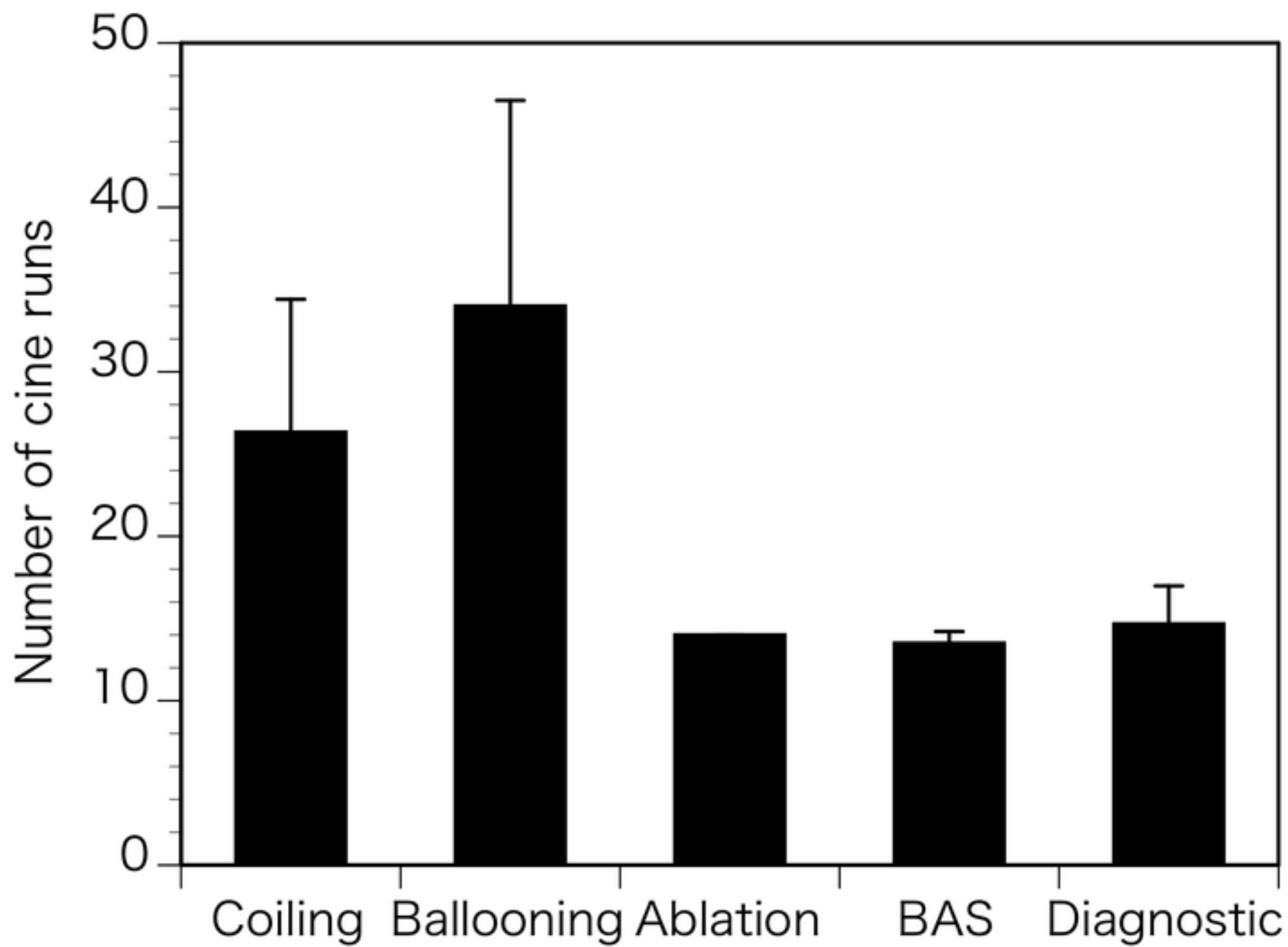

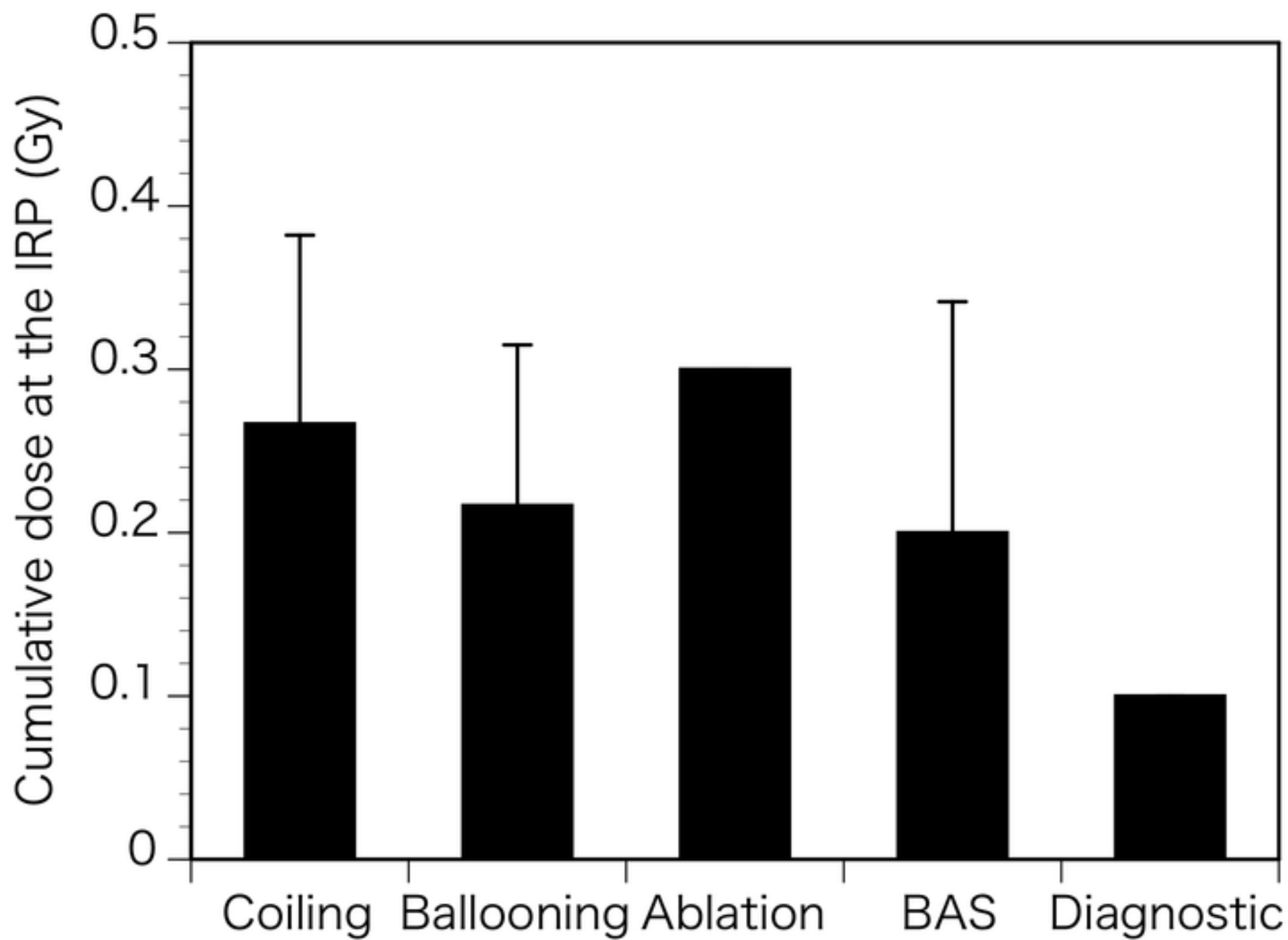

Supplement: Supplementary Data [file supp_rru050_rru050supp_fig1.pdf]
