# Supplementary material for: Direct measurement of a patient's entrance skin dose during pediatric cardiac catheterization
Source: J Radiat Res. 2014 Jun 26;55(6):1122–30. doi: 10.1093/jrr/rru050 (PMC4229915; doi:10.1093/jrr/rru050)
Supplement: Supplementary Data [file supp_rru050_rru050supp.doc]

Supplemental Fig. 1. Angiographic parameter in different procedures. (A) DAP (mean ± SD) in each procedure. 4889.7 ± 3242.4 for coiling (n = 3); 2693.8 ± 576.9 for ballooning (n = 6); 2364.1 ± 0.0 for ablation (n = 1); 1691.1 ± 951.4 for BAS (n = 2); 873.7 ± 487.8 for diagnostic (n = 3). (B) Total number of frames (mean ± SD) in each procedure. 2100.3 ± 777.8 for coiling (n = 3); 3508.7 ± 1593.4 for ballooning (n = 6); 50.0 ± 0.0 for ablation (n = 1); 563.0 ± 725.5 for BAS (n = 2); 1335.3 ± 586.6 for diagnostic (n = 3). (C) Total fluoroscopic time (mean ± SD) in each procedure. 3608.0 ± 1986.0 for coiling (n = 3); 3335.8 ± 1250.3 for ballooning (n = 6); 3530.0 ± 0.0 for ablation (n = 1); 4242.0 ± 4112.5 for BAS (n = 2); 1683.3 ± 1292.0 for diagnostic (n = 3). (D) Number of cine runs (mean ± SD) in each procedure. 26.3 ± 8.1 for coiling (n = 3); 34.0 ± 12.5 for ballooning (n = 6); 14.0 ± 0.00 for ablation (n = 1); 13.5 ± 0.7 for BAS (n = 2); 14.7 ± 2.3 for diagnostic (n = 3). (E) Cumulative dose at the IRP (mean ± SD) in each procedure. 0.27 ± 0.12 for coiling (n = 3); 0.22 ± 0.10 for ballooning (n = 6); 0.30 ± 0.00 for ablation (n = 1); 0.20 ± 0.14 for BAS (n = 2); 0.10 ± 0.00 for diagnostic (n = 3). Data on the graph indicate mean + SD.
